# Invisible borders in educational technology research? A comparative analysis

**DOI:** 10.1007/s11423-023-10195-3

**Published:** 2023-02-07

**Authors:** Victoria I. Marín, Katja Buntins, Svenja Bedenlier, Melissa Bond

**Affiliations:** 1 Universitat de Lleida, Lleida, Spain; 2 University of Duisburg-Essen, Essen, Germany; 3 Friedrich-Alexander-Universität Erlangen-Nürnberg, Fürth, Germany; 4 University of South Australia, Adelaide, Australia

**Keywords:** Educational technology research, Comparative research, Bibliometric analysis, Educational research traditions

## Abstract

Educational research is reflective of the nature and structure of national and regional education systems and their historical evolution. Educational technology research, as an area within educational research, reflects this case particularly prominently. Although individual countries and regions have varying research traditions, the publication of research in English as the scientific *lingua franca* can lead to missing nuances in terminology, which is often not reflected upon. Despite this, the exploration of research from different countries can still uncover diverse topical clusters. This study aims to identify the research topics in educational technology research in three countries (Germany, Spain and the United Kingdom), each with their own research traditions, through the terms used. To this end, a bibliometric analysis of 3034 article abstracts and keywords from 29 English-language Web of Science journals in the field of education and educational research was conducted, with a focus on educational technology. In addition, the quantitative findings are comparatively analysed by considering the corresponding cultural clusters. Main findings include diverse research foci in the three countries, also showing that distinct research traditions are still present, despite using English as *lingua franca*. Therefore, research articles written in English by non-English authors often do not reflect the same meanings in each country, despite using the same words. The conclusions reflect upon the need to establish ways of understanding the traditions behind those research articles and build collaborative systems to illustrate nuances in this research.

## Introduction

Research in education focuses on practices and discourses that are closely interrelated with the understanding and the idea of education, inscribed in national education systems and their historical development. As Berliner (2002, p. 18) argues, educational research is a “hard-to-do science,” since “we face particular problems and must deal with local conditions that limit generalisations and theory building”. The author highlights three issues that affect educational research: “the power of context, the ubiquity of interactions, and the problem of ‘decade by findings’ interactions” (Berliner, 2002, p. 18). These difficulties and issues also apply to research revolving around Educational Technology (EdTech), a now established discipline in educational research around the world, last defined by the Association for Educational Communications and Technology (AECT) as “the study and ethical practice of facilitating learning and improving performance by creating, using, and managing appropriate technological processes and resources” (AECT, 2008, p. 1).

Terminology that is used in EdTech research to describe central topics and constructs varies across countries (e.g., Mayrberger & Kumar, 2014), carrying different meaning and being rooted in specific research traditions and research contexts. This fact and complexity may be one of the causes of the existence of a ‘semantic jungle’ or a ‘Tower of Babel’ syndrome in the field (García Aretio, 2020; Guri-Rosenblit & Gros, 2011). There are very few initiatives that have addressed the complexity of terminology in EdTech, that go beyond individual reflections. For instance, the Slovakian Science Project developed a translation vocabulary of EdTech neologisms in English terminology, as a way to ensure the use of terms in the Slovakian national language are comparable in meaning to the English terms (Mihalko et al., 2003).

Increasingly international scientific communication and exchange due to the reliance on the English language, and the ease of access to globally relevant information and pressing research topics due to digital publishing (e.g., for English language use: Tardy, 2004) are two current phenomena happening in research. Needless to say, research on EdTech is not exempt from this development (Bond et al., 2019). However, resistance has formed against this pattern, arguing that no uniformity in educational research interests exists and that alongside cultural differences and linguistic diversity, publishing solely in English fails to realise the aims of educational research (Ruiz-Corbella et al., 2014).

Therefore, in this article we argue that, despite publishing in the same English-language journals, EdTech researchers from different countries continue to pursue distinct research topics and ground them in different theoretical backgrounds, leading to country-specific topical clusters, as previous research tentatively suggests (e.g., for the context of Spain, see Marín & Zawacki-Richter, 2019). This translates into questions about what topics researchers from different countries focus on and which terminology they use to describe their research.

The current bibliometric study provides a descriptive overview of the distribution of research topics and author-chosen terminology within EdTech research, and frames the country-specific discussions within cultural clusters and research traditions, to foster critical dialogue on the seemingly homogeneous international research community versus country-specific traditions and topics, and the prevalence of certain research disciplines over others.

## Research traditions in EdTech

In order to understand the different research traditions in the field of EdTech, the cultural clusters delineated by Ronen and Shenkar (2013) are of particular value. The authors argue that national culture greatly influences differences between countries and, hence, suggest that clustering can guide cross-cultural sampling and avoid biases in measuring cultural differences. Based on an eco-cultural framework that considers three main variables (religion, language and geography), the authors identify 11 global clusters: Arab, Near East, Latin America, East Europe, Latin Europe, Nordic, Germanic, African, Anglo, Confucian and Far East (Ronen & Shenkar, 2013).

The three countries selected for this study—Germany, Spain and the United Kingdom—have established research traditions in the field of education research. Biesta (2011) traces the two major lines as the Anglo-Saxon tradition from curriculum studies, and the continental tradition from *Pädagogik.* In this understanding, Germany and Spain are attributed to the continental tradition and the United Kingdom to the Anglo-Saxon tradition (Biesta, 2011; Castañeda et al., 2020). These traditions have also been influential in the specific area of EdTech research (Castañeda et al., 2020). An additional layer that potentially influences research traditions (indirectly) as well, is the allocation of each of the three countries to distinct cultural clusters that are derived from work-related cultural differences and use of religion, language and geography as ecocultural variables to delineate the respective clusters (Ronen & Shenkar, 2013). Based on these variables, Germany (with Austria and Switzerland) forms the Germanic cluster, Spain (with Portugal, Italy, Belgium, France and French speaking Switzerland) the Latin European one and the United Kingdom (with Australia, Ireland, New Zealand, USA, Canada) constitutes the Anglo cluster.

### Germany: Medienpädagogik

As Biesta (2011) shows, education science in Germany is conceived in the tradition of *Pädagogik* in the aforementioned continental understanding. For the research and practice along the nexus of (digital) media, learning and education, subjectivation processes and socialisation—to name but a few central concepts—the term *Medienpädagogik* is used. With *Medienpädagogik* now being considered as a subfield within Education Sciences, it is roughly divided into *Medienerziehung* (media education) and *Mediendidaktik* (media didactics) (Kerres, 2018)—although it must also be acknowledged that a translation can only roughly consider the underlying concepts and attached meanings. Following Kerres’ (2018) differentiation, both are interrelated, with *Medienerziehung* revolving around the conception of the active subject dealing competently with media, whereas *Mediendidaktik* focusses on the design of media environments conducive to learning processes. However, this rather clear-cut differentiation is not undisputed, with Swertz et al. (2017) asking the question whether *Medienpädagogik* is a scientific discipline of its own, maybe a subdiscipline of education sciences, communication studies, informatics or media studies, or rather an inter/multidisciplinary field. Characteristic of *Medienpädagogik* is its constitution and orientation towards operating at the interface of research and practice, thus, to closely observe media practices and subjective appropriation processes (Schiefner-Rohs, 2013).

The scientific community encompasses researchers mainly based in Germany, Austria and Switzerland, and thus follows linguistic proximity and shared underpinnings in theory development. It also needs to be noted that three central scientific journals for the community are each hosted in one of the three countries: *Medienimpulse* (Austria), *Medienpädagogik* (Switzerland) and *medien* + *erziehung* (Germany).

### Spain: Tecnología Educativa

In Spain, the research field is commonly known as *Tecnología Educativa*. Its origins relate to audiovisual education (Martínez Sánchez, 2008) and the introduction of programmed learning (García-Valcárcel, 2003). The classic *Tecnología Educativa* has been framed in the national context within *Didáctica*, which is one of the applied education sciences and is concerned with teaching and learning processes (García-Valcárcel, 2003). Therefore, the field (and practice) of *Tecnología Educativa* builds upon a more ‘continental European’ pedagogical tradition (*Pädagogik)*, but it is also founded in three imported Anglo-Saxon pillars (psychology, communication theories and general systems theory) (Castañeda et al., 2020). *Tecnología Educativa,* as a research field in its own right, is usually found in Spanish university *Didáctic*a departments, as a discipline with specific research lines framed within the education sciences and *Didáctica* (Cabero, 2007; García-Válcarcel, 2003). In some universities it is found in *Pedagogía* or Education Sciences departments. The two classic main areas are *Tecnología educativa* understood as instructional design (design of technology mediated learning situations) and therefore equivalent to one of the theoretical approaches of *Didáctica*, and *Tecnología educativa* as the study of media from non-instrumental positions, as a discipline situated within *Didáctica* (García-Valcárcel, 2003).

These theoretical underpinnings can be identified through the evolution of the field in Spain and other Spanish-speaking countries. This evolution is represented, for instance, by the studies published in the journal EDUTEC, which is the outlet of the main association in Ibero-American countries for EdTech. In an analysis of EDUTEC’s publications, Marín et al. (2017) delineated a first phase (1995–2004) of reflections on the educational opportunities of technologies, with a special focus on communication and communication tools (continental and Anglo-Saxon traditions). The second phase (2005–2015) identified a focus on how to improve learning (and teaching) with technologies, alongside the relevance of developing students’ (and educators’) digital competences (continental and national traditions). Similarly, the analysis of the periods where the *Journal of Educational Technology in Higher Education* published articles in Spanish under the title *RUSC. Universities and Knowledge Society Journal* identified two periods: (1) reflections and studies on the use of ICT in higher education (2004–2009) and (2) the quality of learning using technologies (2010–2015) (Marín et al., 2018). Another bibliometric analysis on a different Spanish journal (RELATEC) revealed the integration of technologies (policies, program and project evaluation), attitude towards/acceptance of technologies, learning environments and interactive learning, and online learning, as the main research topics across studies published between 2002 and 2017 (Rodríguez-Miranda & Bolaños, 2018).

Against this backdrop, the research field of EdTech in Spain is a rather eclectic and dynamic case in its theoretical underpinnings, which is worthwhile exploring.

### United Kingdom: EdTech and technology enhanced learning (TEL)

In the United Kingdom (UK), terminology describing the field of EdTech has certainly changed throughout the past fifty years, despite the presence of a preeminent journal with that title (*British Journal of Educational Technology*; see BJET Editors, 1970). The analysis of EdTech publications across 50 years in BJET by Bond et al. (2019) uncovered the use of ‘media’ and ‘multimedia learning’ in the 1970s, predominantly as a result of research exploring radio and television broadcasting (e.g., Scupham, 1970), as well as telephone conferencing and the integration of media into courses (e.g., Bates & Pugh, 1975). The 1980s saw a transition from audiovisual to more computer-based learning and ‘computer-assisted instruction’ (Barker & Singh, 1983). It was not until the 1990s that the phrase ‘interactive multimedia’ overtook the use of the word ‘media’ (Plowman, 1996), alongside the use of email and the internet (e.g., Pitt, 1996) and a growing movement towards constructivist and collaborative approaches to support learning (Laurillard, 1995).

In the ‘noughties’ (2000–2009), the phrases ‘virtual learning environments,’ ‘learning technology’ and ‘e-Learning’ became popular. This was followed by the rise of ‘technology enhanced learning’ towards the 2010s (Bayne, 2015) as a result of national policy changes, with TEL becoming the *lingua franca* of the Universities and Colleges Information Systems Association (UCISA, 2008) and the UK higher education system at large (Kennedy & Dunn, 2018). Proponents of TEL argued that it required a more structured and nuanced understanding of learning design, and greater consideration of learner needs (e.g., Laurillard et al., 2009), reflected in a recent systematic review of TEL Masters programs (Fominykh et al., 2022). However, criticism was levelled at the “unconsidered and unreflected way” (Kirkwood & Price, 2014, p. 9) in which it was used, with questions posed about its “effective educational contribution” (ibid., p. 26). It is now considered an interdisciplinary research field that has continued its growth in application outside of formal higher education, including health (e.g., Health Education England’s Technology Enhanced Learning Programme; NHS, 2022).

## Research questions

Against this background, two research questions are addressed in this study:

- Which terminology do researchers from Germany, Spain and the UK use to describe their research in EdTech?
- What EdTech research topics do authors from these three countries focus on?

## Method

Bibliometric and content analyses of scientific EdTech journals can serve to identify overarching research topics over time, as well as show the distribution of contributions across individual countries (e.g., Bond et al., 2019; Marín et al., 2017). Still, previous analyses have not yet thoroughly investigated whether research topics are also distinct for authors from different countries. Bibliometric analysis has, in conjunction with topic modelling, emerged as a suitable approach to delineate and quantify research topics in journal publications (e.g., Bond & Buntins, 2018).

In order to answer the research questions of the present study, a bibliometric analysis was conducted using the Bibliometrix package in R (Aria & Cuccurullo, 2017). Abstracts, author keywords and references were retrieved from 29 journals in the English language, indexed in the Web of Science database (Education and Educational Research). As a proxy for the scope of EdTech, journals were included whose titles included terms such as *educational technology*, *computers*, *technology enhanced*, but also well-known journals, such as *Distance Education*. For this analysis, we consider a total of 3034 articles originating from authors with institutional affiliations either in Spain, Germany or the UK and covering the time period from 2012 to 2021.

The Bibliometrix package offers the function of extracting authors, research location, references and keywords from the metadata of literature databases. The different data points can also be displayed in networks. For the evaluation, we checked the data for consistency. Authors' keywords were randomly checked for correct separation and spelling.

Subsequently, the keywords were clustered into categories. For this purpose, the first 200 keywords per country were evaluated and categorised. This resulted in 21 different categories. These were extracted into separate data sets. Further comparable keywords were identified in the search. Examples of authors’ keywords are shown in Table 1.

**Table 1:** Categories with examples of authors’ keywords

Categories	Keyword 1	Keyword 2	Keyword 3
EdTech Basic Terms	E-Learning	Technology	Online Learning
Learning Process	Student Engagement	Self-regulated learning	Motivation
Tools	Mobile learning	VR	Video games
Collaboration	Collaborative learning	Collaboration	Computer-mediated communication
Research Methods	Evaluation	Eye tracking	Thematic Analysis
Pedagogy/ Teaching Approaches	Improving Classroom Teaching	Pedagogical Issues	Teaching/Learning Strategies
Outcome	Assessment	Feedback	Telematic performance
Not codable/ not EdTech	Innovation	Health	Privacy
Teaching subject	Mathematics	Music Technology	Teacher Education
Educational Sector	Higher Education	Informal Learning	Secondary Education
Basic Terms	Education	Learning	Teachers
Covariables	Gender	Age	Individual differences
UX	Usability	User Experience	Accessibility
Social Media	Social Media	Facebook	Social networking
Problems	Internet addiction	Cyberbullying	Risk
Big Data & Personalisation	Learning Analytics	Artificial Intelligence	Machine Learning
Country/Cross-Culture	Cross-cultural	South Africa	Country-specific developments
Age	Adolescents	Teenager	Adult
Labour Market	Human Factors	Employability	Leadership
Hardware	Smartphones	Mobile Devices	Cloud Computing
Time period	Covid-19		

## Results

Out of the 3034 abstracts under consideration and taking into account that authors of the two or three countries could be present in the same article, 826 articles were written by Germany-based researchers, 1007 originated from Spain and 1339 were published by researchers from the UK, making the latter country the dominant one. Abstracts from Spanish and German authors were sourced from 27 English language EdTech journals, while abstracts with UK authors were sourced from 29 journals.

### Basic data

The journal featuring the most EdTech articles across all three countries is *Computers in Human Behavior*, a journal focusing on the use of computers from a psychological perspective. The following journals in the ranking differ from each country, although *Computers & Education* has an important share of the publications in our sample across the three countries (see Table 2 for the ranking and Appendix 1 for the complete list with the percentages per sample).

**Table 2:** Top 10 publication outlets in the three countries

Journals	Germany	Spain	UK
Computers in Human Behavior	1	1	1
Computers & Education	2	2	3
British Journal of Educational Technology	6	3	2
IEEE Transactions on Learning Technologies	4	4	7
Journal of Computer Assisted Learning	3	10	5
Education and Information Technologies	8	6	4
Educational Technology & Society		7	10
ETR&D-Educational Technology Research and Development	7		
International Journal of Educational Technology in Higher Education	10	5	
International Journal of Emerging Technologies in Learning		9	
International Journal of Mathematical Education in Science and Technology		8	6
Internet and Higher Education			9
Distance Education			8
Eurasia Journal of Mathematics Science and Technology Education	9		
International Journal of Computer-Supported Collaborative Learning	5		

In the case of the United Kingdom, the *British Journal of Educational Technology* is also prominent (15.2%) and present, to an important degree, in the other two countries (Germany: 3.8%; Spain: 7.4%). With a similar share, German authors publish in the *Journal of Computer Assisted Learning* (5.3%) and UK authors in the *Education and Information Technologies* journal (5.2%). *IEEE Transactions on Learning Technologies* and the *International Journal of Educational Technology in Higher Education* seem more popular among Spanish authors than in the other countries (7.1% and 5.4%, respectively). All these journals are in the Q1 of the Web of Science ranking within Education & Educational Research.

UK authors were the most likely to collaborate with authors from other countries, with 69.5% corresponding authors from the UK in the articles in our sample and the rest from other countries (e.g., China, Australia, USA). In the case of the German sample, 72.8% of the corresponding authors were from Germany, with collaborators from the Netherlands, USA and UK, among others. Finally, the Spanish sample shows that 81.9% were Spanish corresponding authors; and therefore, the sample is the one out of the three with less international collaborations (e.g., with the UK, Chile and Colombia).

The 10 most productive authors in each country are shown in Table 3. This overview allows us to consider the disciplinary background of authors who shape publications in English EdTech outlets. For instance, in the case of the authors affiliated with Spanish institutions, 8 out of the 10 come from disciplines related to computing (e.g., computer science, software engineering). In contrast, the top 10 Germany-based authors are almost exclusively working in the field of educational psychology, and at the nexus of educational technology.

**Table 3:** Most productive authors

Rank	UK	Spain	Germany
1	Gasevic D	Hernandez-Leo D	Rey GD
2	Griffiths MD	Alario-Hoyos C	Cress U
3	Rienties B	Delgado Kloos C	Ifenthaler D
4	Dawson S	Asensio-Perez JI	Schneider S
5	Kaye LK	Daradoumis T	Nebel S
6	Littlejohn A	Munoz-Merino PJ	Nistor N
7	Herodotou C	Ordonez De Pablos P	Weinberger A
8	Joksimovic S	Perez-Sanagustin M	Hesse Fw
9	Marder B	Barbera E	Fischer F
10	Tsai Ys	Fernandez-Manjon B	Kraemer NC

### Which terminology do researchers from Germany, Spain and the United Kingdom use to describe their research?

Across the three countries, results indicate that research revolves around *learning* (first top keywords in Germany and the UK, and second one in Spain) *and higher education* (first top keyword in Spain and second one in Germany and the UK) as the two most frequent terms (see Table 4).

**Table 4:** Frequency of *learning* and *higher education* as keywords across the three countries

Country	Learning (f)	Higher education (f)
Spain	65	76
Germany	65	62
United Kingdom	45	30

Aside from these common terms, research from the three countries is characterised through very heterogenous terms. Nevertheless, across all three countries there is also a discernible focus on collaboration and collaborative learning, the study of motivation, and a research interest in learning analytics.

For Germany-based studies, terms such as *social* and *social media* are frequent, as are terms such as *multimedia learning*, *cognitive load*, *self-efficacy* and *eye tracking* that make reference to educational psychology research, rather than relating to topics addressed in the field of pedagogy. The term *Media in Education* is likewise among the top 20 keywords but does not appear in the top 20 of the Spanish and UK samples.

In contrast, abstracts with Spanish origin indicate terms that connect to technologies and strategies related to gaming. For instance, among the 20 keywords we can identify *augmented reality, gamification* and *virtual reality*. However, the use of relevant concepts or ideas related to pedagogy and education is noticeable too, such as *teaching/learning strategies* or *improving classroom teaching*, and the research interest in *cyberbullying* and *adolescents*. It is noteworthy that *assessment* is among the 20 top keywords, and that it does not appear in the other samples.

In UK-based studies, the top 20 most common keywords also refer to *social media*, *education* and *learning analytics*. The most common UK keywords also revealed the move towards *collaborating* online and the rise of *mobile learning*. Particularly interesting here, however, is a reflection on research that explored the *social* effects of technology, such as *internet addiction*, with terms addressing so-called ‘problems’ featuring higher in UK research than in that from Spain and Germany.

### What research topics do authors from these three countries focus on?

**Fig. 1:**
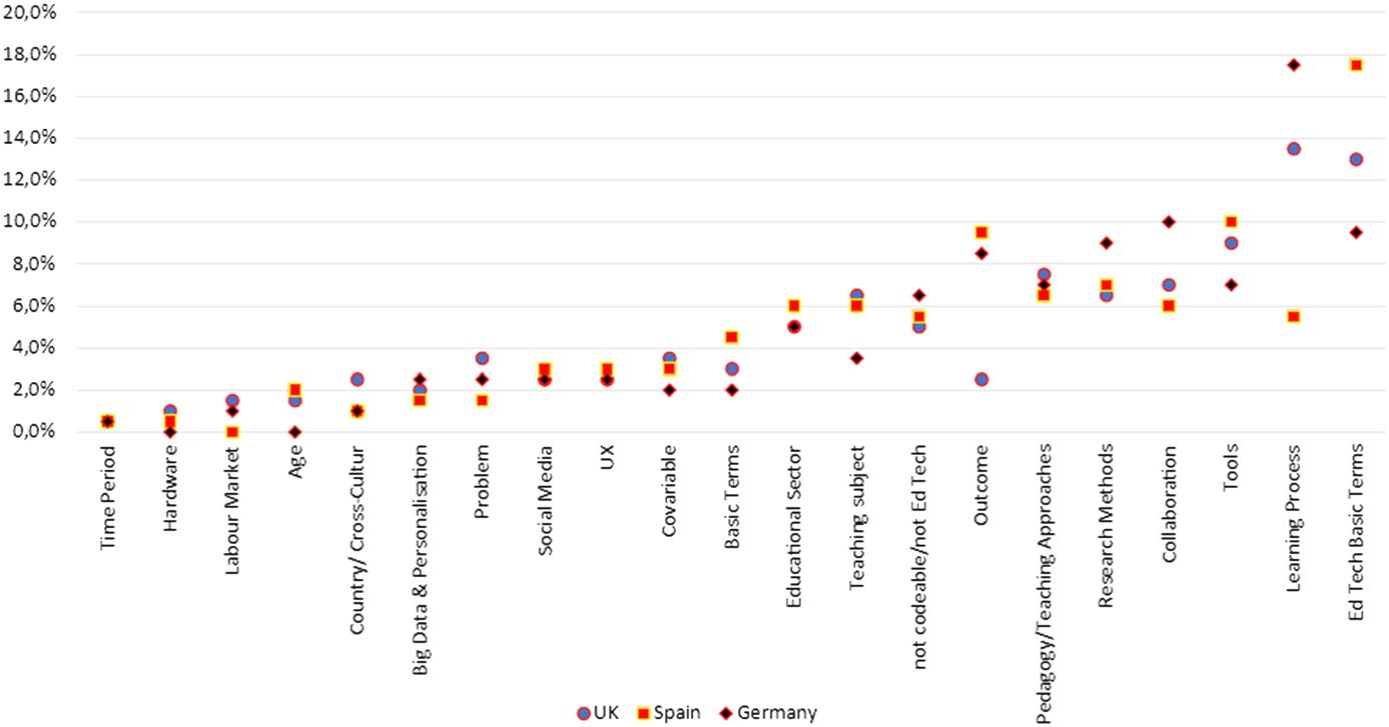
Comparison of groups of research topics between the three countries

Keywords were clustered according to different aspects in order to compare their presence across the countries (see Fig. 1). Then, the most prominent groups in each country were analysed to distinguish the most representative research topics. The analysis is based on the 200 most frequent keywords in each country. For the percentages, each entry is weighted equally, i.e., only the occurrence and not the frequency of occurrence is counted.

Research topics emerged as distinct for each country, covering a range of topics and even indicating specific communities within countries. Despite commonly shared terms, research foci deviate considerably.

#### Germany

**Fig. 2:**
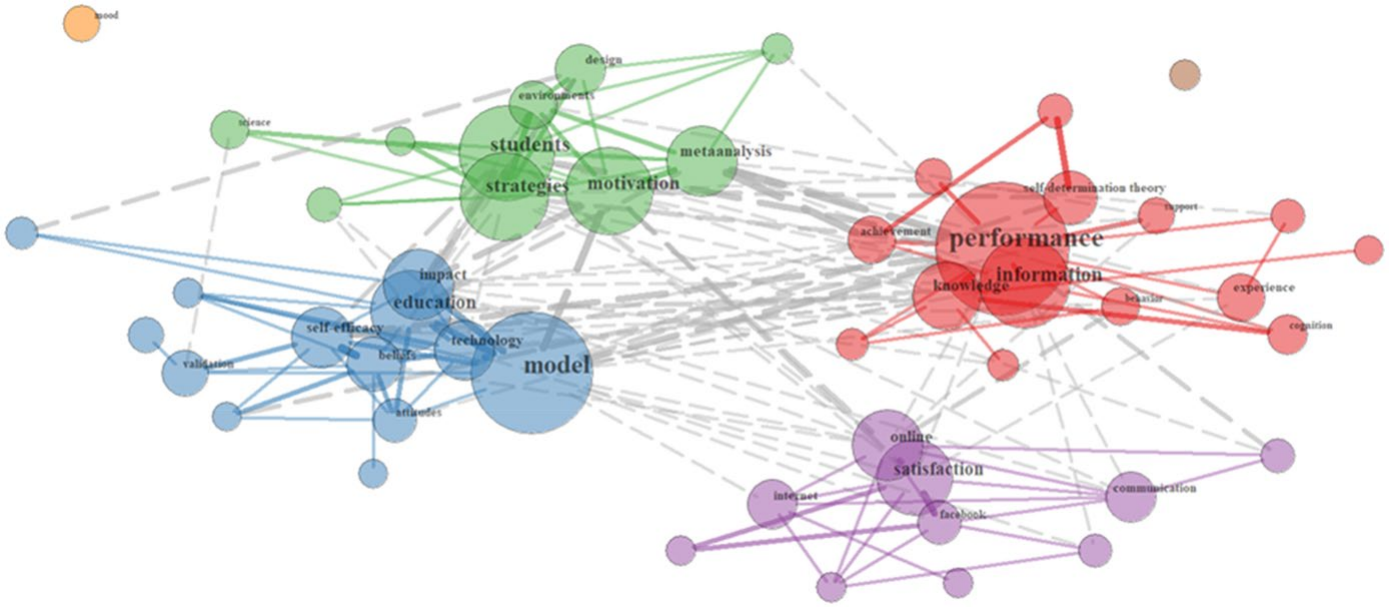
Relationships of Learning process terms in the German sample

In the German sample, keyword clusters that revolve around the *learning process* (17.5%), *collaboration* (10%) and *research methods* (9%) stand out. Focusing on the *learning process*, it is terms such as *cognitive load, motivation, self-efficacy, self-regulated learning* and *metacognition* that illustrate primarily topics related to constructs used in educational psychology. As depicted in Fig. 2, these constructs are found to group around self-regulated learning strategies, e.g., *motivation, strategies, environments, students* (see green group) but also revolve around *self-efficacy, beliefs, attitudes* in relation to *education* and *technology* (see blue group). Other terms with an important presence are *performance* (see red group) and *satisfaction* (see purple group).

**Fig. 3:**
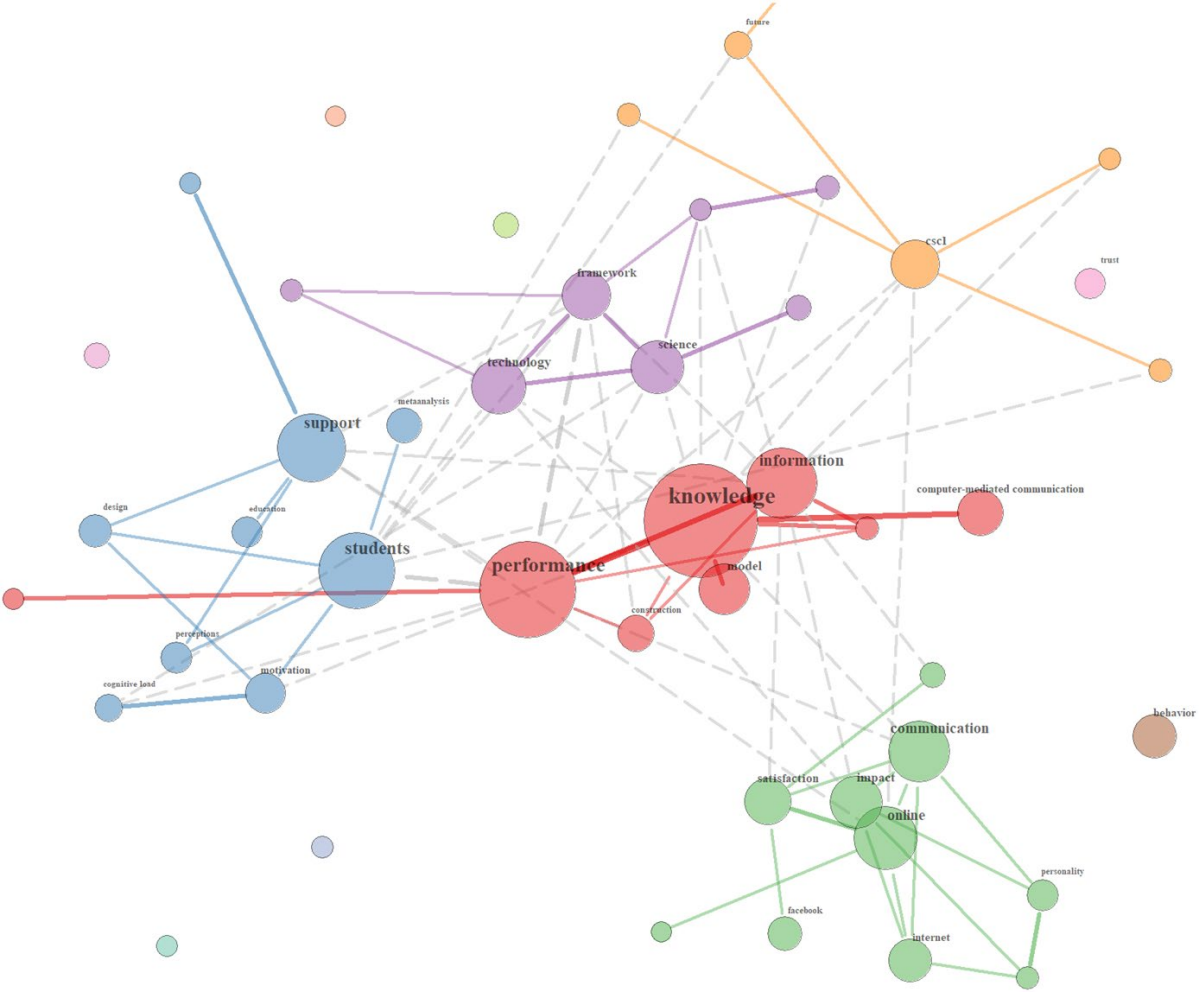
Relationships of Collaboration terms in the German sample

For the cluster c*ollaboration,* concepts from educational psychology are likewise prominently present. One group connects around the topics of *knowledge*, *information* and *performance* (see red group), a second one revolves around *communication* and also includes terms such as *impact*, *personality* and *satisfaction* (see green group). A third group centres around *students* and *support*, while also considering *design*, *motivation* and *cognitive load* among others (see blue group). Thus, with collaboration as an overarching term, distinct subthemes emerge (see Fig. 3).

**Fig. 4:**
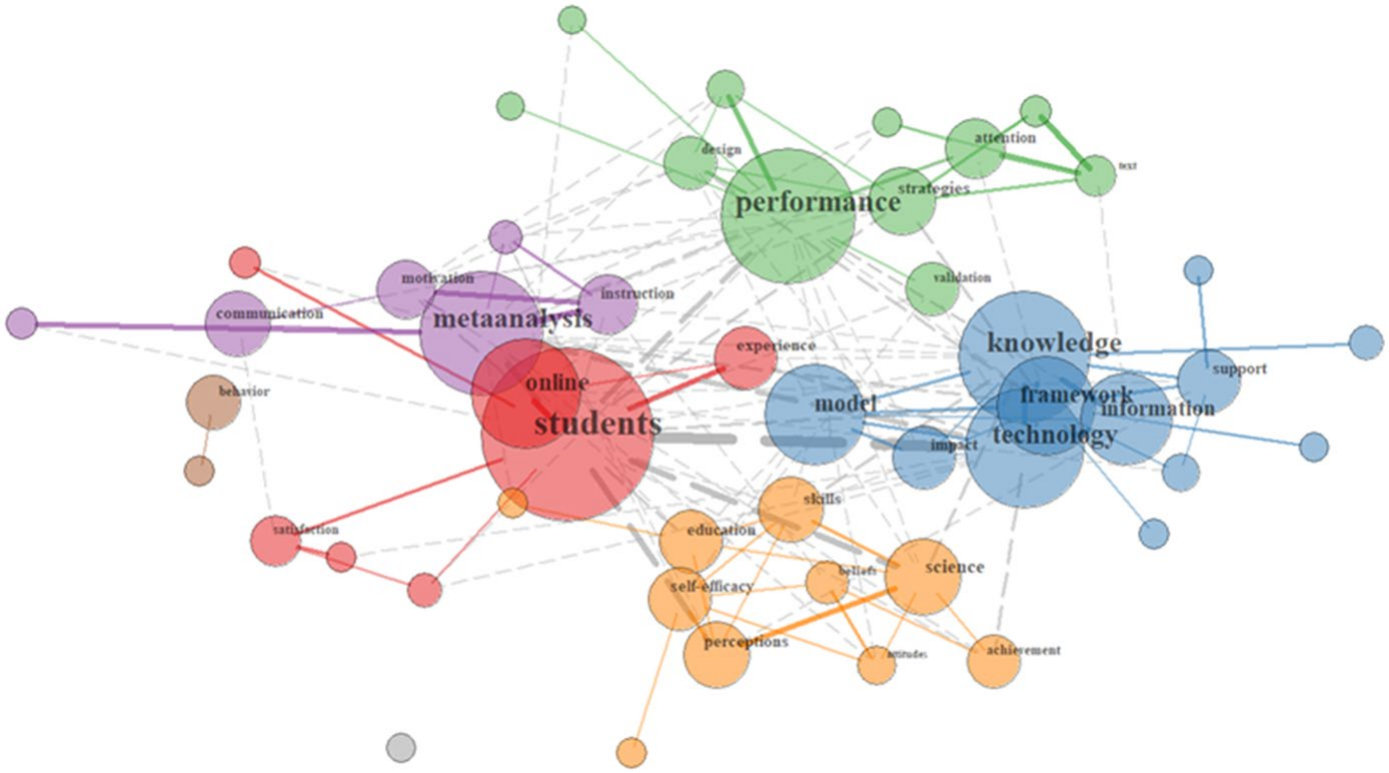
Relationships of Research Methods terms in the German sample

The educational psychology focus is most pronounced in the third cluster, that is the one centred around *research methods* (see Fig. 4). To highlight this, *metaanalysis* forms the node of one group (the purple one), with *performance* being the most prominent concept connected directly with *validation* in another group (the green one). Another group unites central topics of education psychology research, including *achievement*, *self*-*efficacy*, *attitudes* and *skills* (see orange group).

#### Spain

**Fig. 5:**
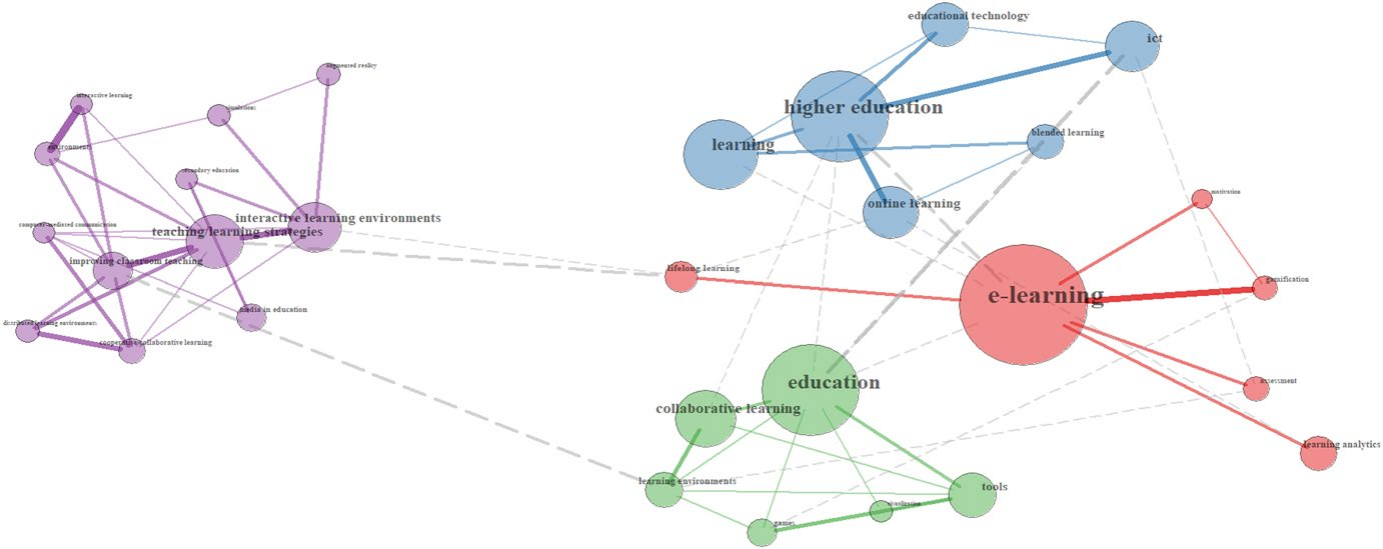
Relationships of EdTech Basic Terms in the Spanish sample

The three prominent clusters of keywords in the Spanish sample are *EdTech basic terms, tools and outcomes*. Among Ed Tech basic terms (17.5%) a broad terminology that refer to digital tools and education can be found (e.g., *e-learning, ICT, blended learning, online learning*). These terms, in relation to *teaching/learning strategies* (purple group), *higher education* (blue group) and *collaborative learning* (green group), delineate different main research topics and contexts in this sample (see Fig. 5). *Motivation, gamification, assessment* and *learning analytics* (red group) also appear as research interests in connection with e-learning, though with lesser presence.

**Fig. 6:**
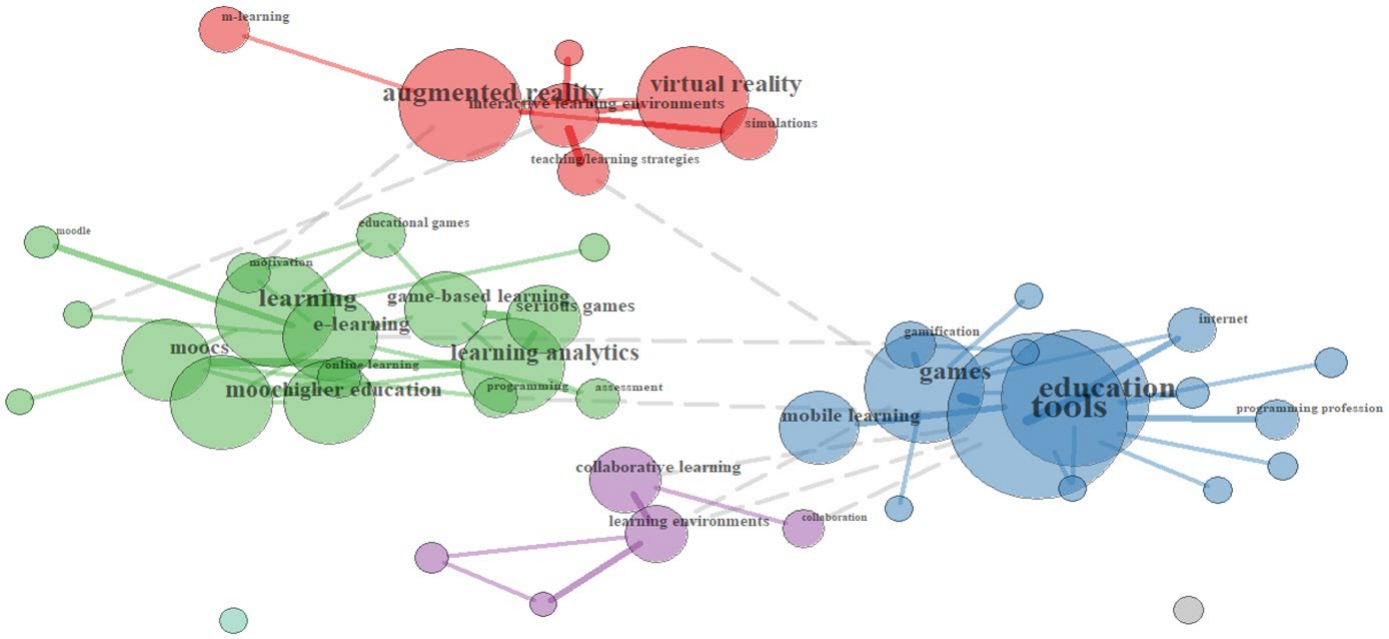
Relationships of Tools terms in the Spanish sample

The second cluster concerns *Tools* (10%). Here, many different terms related to technologies and platforms are present (e.g., *interactive learning environments, MOOCs*), and especially those related to gaming or gaming-like environments (e.g., *virtual reality, augmented reality, games, serious games, educational games*). Also, *mobile learning and m-learning* appear in relation to gaming-like apps. These terms represent different research interests in Fig. 6, with *games* as *tools* used in *education* as *mobile learning* (see blue group), the application of different *interactive learning environments* (*augmented reality, virtual reality, simulations, with m-learning*) to implement specific *teaching/learning strategies* (see red group) and a mix of different topics in *higher education* such as *MOOCs* and *learning analytics*, but also *game-based learning* or *serious games* (see green group). The purple group, with lesser presence, refers to *learning environments* for *collaborative learning*.

**Fig. 7:**
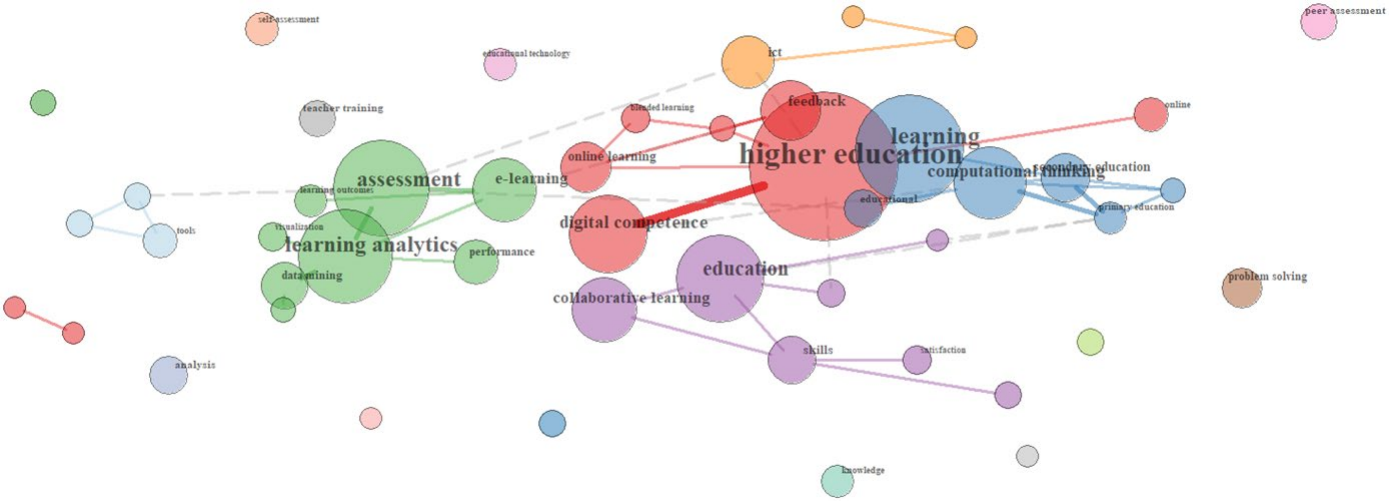
Relationships of Outcomes terms in the Spanish sample

Finally, in the case of the cluster *Outcomes*, research interests connected to *assessment and learning analytics* (see green group)*, feedback and digital competence in higher education* (see red group), and *computational thinking* (see blue group) are highlighted (see Fig. 7). *Collaborative learning* has some presence too (see purple group).

#### United Kingdom

**Fig. 8:**
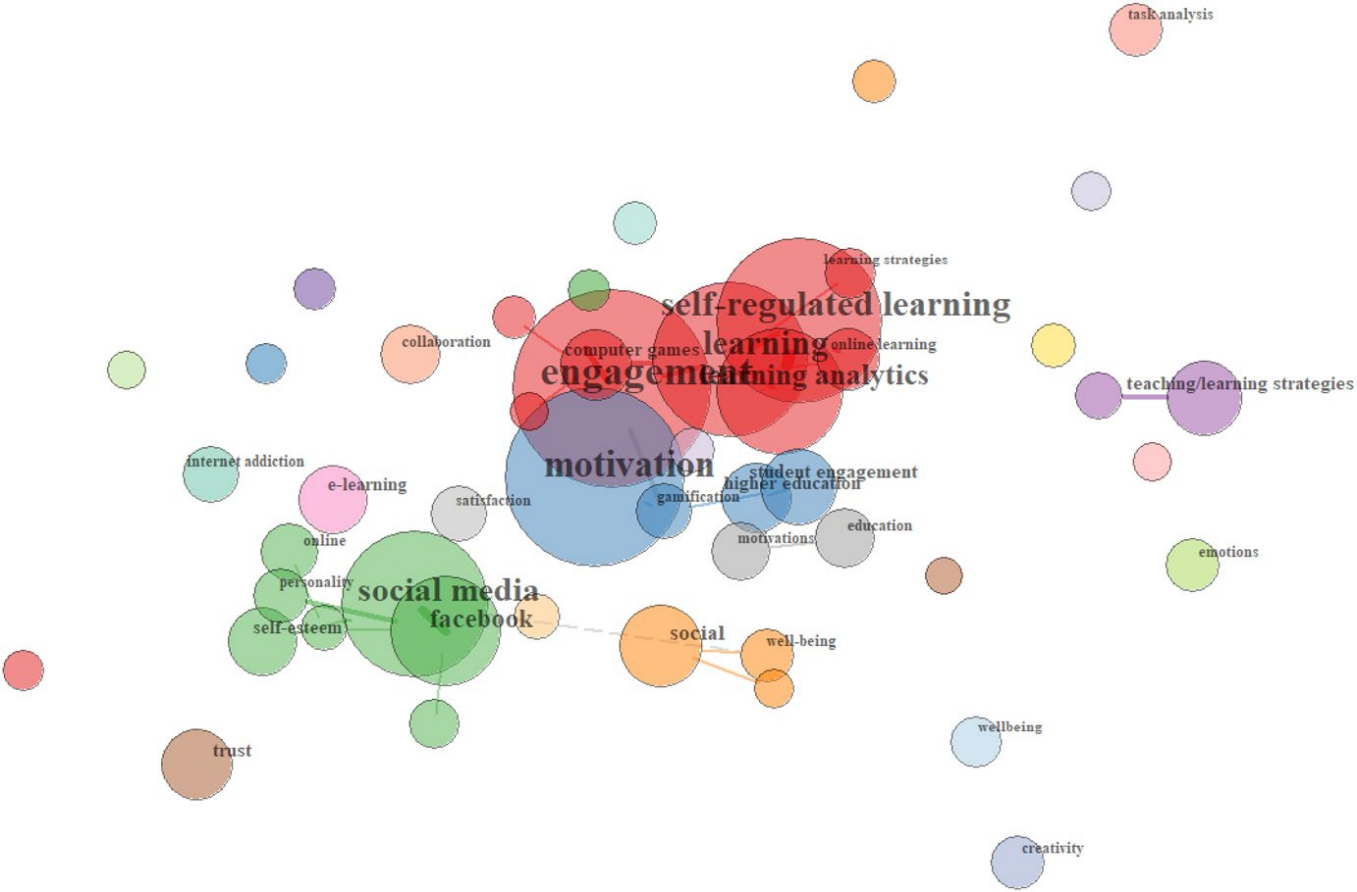
Relationships of Learning process terms in the UK sample

For the UK case, the picture is different again. The bibliometric analysis of the most frequently used terms under the heading of *learning process* (see Fig. 8), which were found in almost 14% of all UK research in the sample, reveals that researchers were particularly exploring how technology affects *student engagement* and *motivation*, as well as which particular *teaching and learning strategies* could be employed to assist this. Looking at the relationships of the terms coded under learning process, *engagement*, *motivation* and *self-regulated learning* occurred frequently in research on *learning analytics*, and to a slightly smaller extent, *computer games*, *gamification* and *online learning* (see red and blue groups). Research by UK authors also explored the effects of *social media* on *self-esteem* and student satisfaction and well-being, as well as how *social media* could be harnessed to enhance *student engagement* and the expansion of professional learning networks (see green group), as well as the role that mobile apps can play in enhancing *social* interaction (see orange group).

The keywords coded under the heading *EdTech Basic Terms* focused particularly on *e-learning*, *online* and *blended learning*, as well as *distance learning* and *distance education* in particular. Given the focus on collaboration as a key competency for twenty-first century learners (OECD, 2018), there was a substantial focus here on collaboration within online environments, particularly within *higher education*, including the development of online communities within *interactive learning environments* to help facilitate it.

**Fig. 9:**
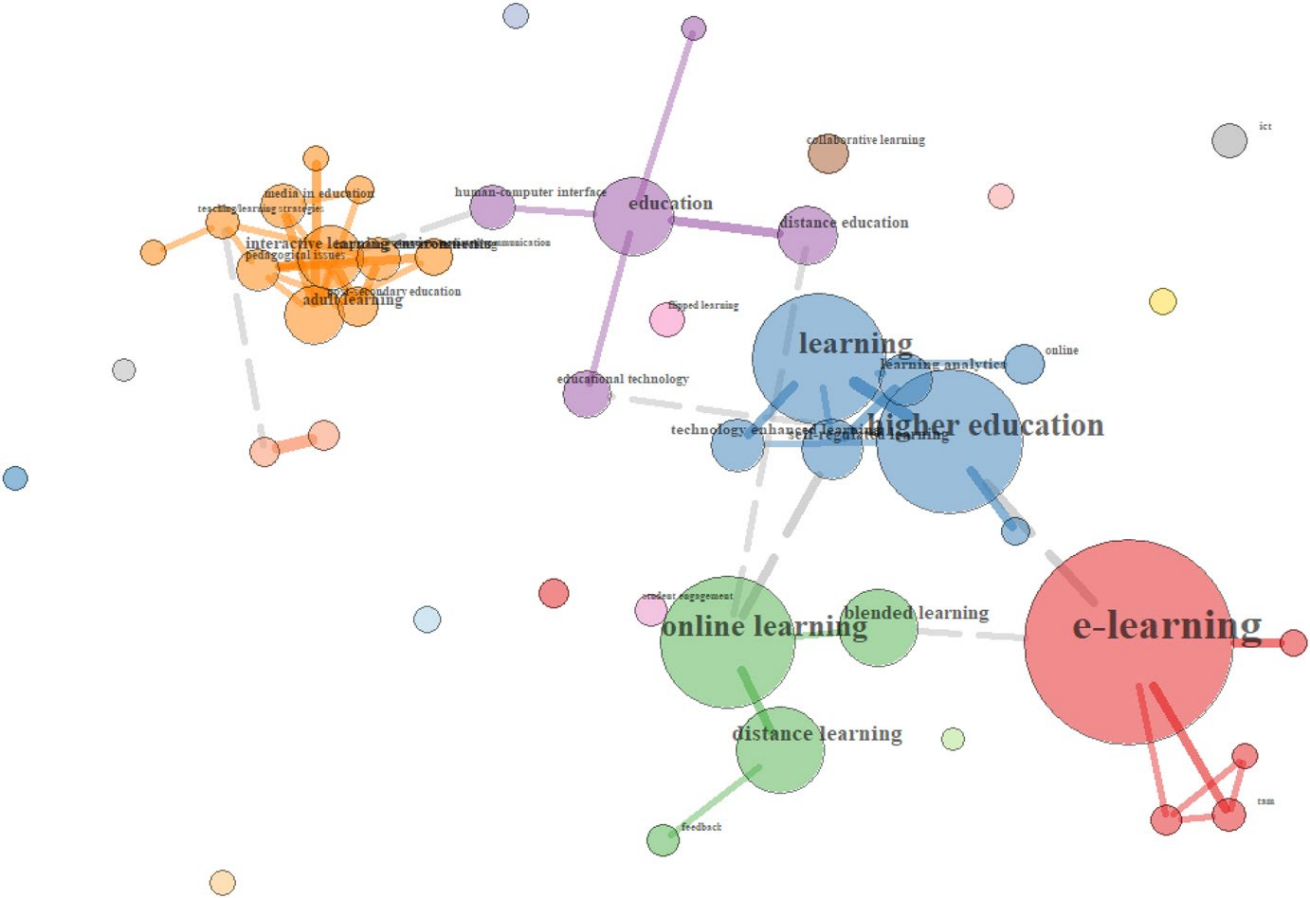
Relationships of EdTech basic terms in the UK sample

Focusing on the relationship of these terms (see Fig. 9), the focus on *learning*, *self-regulation* and *engagement* in *online, distance and blended learning* in *higher education* is particularly evident (see blue and green groups, respectively). The close co-occurrence of the terms *interactive learning environments*, *pedagogical issues*, *teaching/learning strategies*, and *adult learning* can also be observed (see orange group).

**Fig. 10:**
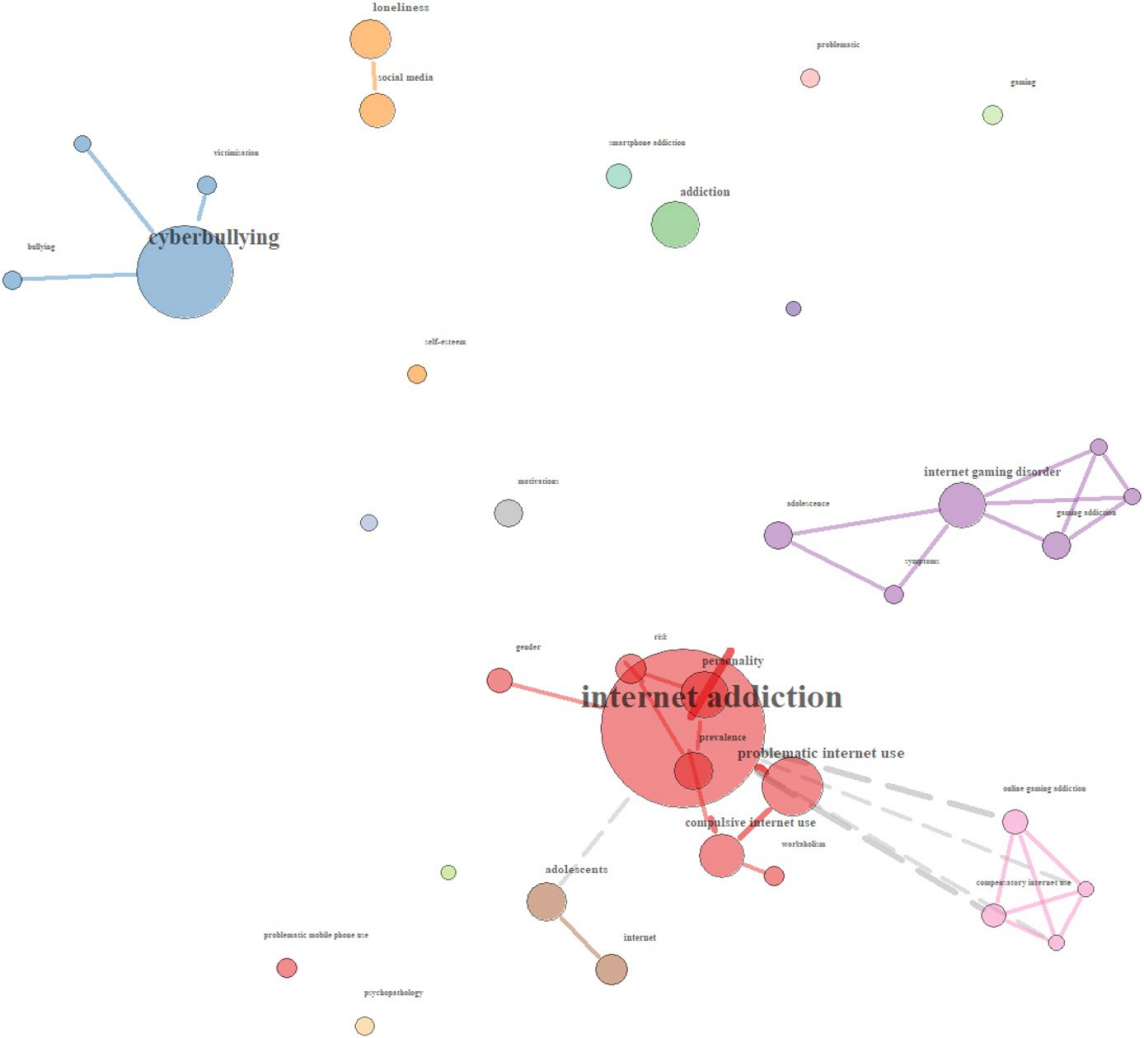
Relationships of Problems terms in the UK sample

Quite unique to the UK sample, was the higher frequency of terms that were coded under the heading of *problems* in the use of technology in education. *Internet addiction* and *cyberbullying* were the two most frequently occurring terms, with research looking in particular at the *prevalence* of *addiction* to various applications and uses of technology, and the psychological harms that stem from them (e.g., *online gaming addiction*, see pink group). This is reflected in the relationships between these terms (see Fig. 10), where the co-occurrence of *internet addiction* with *prevalence* and *adolescents* can be seen (see red and brown groups, respectively). This is also reflected in the purple group of *internet gaming disorder*, symptoms and *adolescence*. Research also explored *cyberbullying* and victimisation (see blue group), as well as *loneliness* attributed to the use of *social media* (see orange group).

## Discussion

The results presented in this study are indicative of the fact that researchers from these three countries do not ‘shed their research community skin’ when they decide to publish in English-language journals. Rooted in distinct research traditions and forming part of a national scientific community that has (simply) switched language, topical clusters are maintained. These results are in line with Biesta (2011), Castañeda et al. (2020), as well as Ronen and Shenkar (2013). Furthermore, Ronen and Shenkar (2013) raise the concept of *convergence* versus *divergence* in relation to their identified cultural clusters. Based on the results of this study, using English as a publication language has not led to a cross-country convergence of research topics or adoption of unequivocal definition and use of terminology.

For Germany-based contributions, it is noteworthy that the term *media in education* appears quite frequently, while it is an unusual term in other countries (Buntins et al., 2018). Still, whereas the term can linguistically be understood, the connotation (e.g., reference to the educational process, educational contexts in general, educational institutions) cannot be sufficiently gathered from any literal translation to German (Mayrberger & Kumar, 2014). In contrast to the multifaceted discussions expressed in German-language outlets and the respective community, the internationally visible discourse in the field of EdTech consists mainly of authors with an overlap to (educational) psychology as well as technology-focused research. This means, however, that a large part of the scientific discourse rooted in the *Medienpädagogik* community, including both content, methodology and methods, is not visible beyond this linguistically delineated community. Relating this to Ronen and Shenkar (2013), the three countries are also considered as one cluster with potentially geography and language being decisive for joint conferences and publication outlets, as well as referring to the continental tradition in education research (Biesta, 2011).

The Spanish sample shows a prominence of EdTech terms and tools, with learning processes less present. This may be related to the background of the authors writing in English in those top journals, who come from disciplines related to computing. This seems to connect with the idea that there are sub-communities inside the same Spanish community; those who write in English in these international journals, and those who do not or do it less frequently. Reasons could be varied and include different promotion systems within disciplines, difficulties with the English language, and relevance to a local/regional/national audience, among others. Previous content analyses of Spanish EdTech journals show the relevance of other education-related terms that were not prominently present in the sample, for example: innovation, educational actors (referring to students, teachers…), educational resources, learning design, quality and so on (e.g., Marín et al., 2017, 2018). Differences in the research interests between the two Spanish sub-communities are also present (e.g., Rodríguez-Miranda & Bolaños, 2018). In addition, in previous research in EdTech journals in English and Spanish language, two different communities with reduced communication among them were acknowledged (Marín & Zawacki-Richter, 2019). This has an important effect on the terminology used in the field, which may include literal translations that do not reflect the same meanings (Castañeda et al., 2020), and missing terms in the internationally visible discourse that are more present in the Spanish-language outlets and the respective community–with their scientific discourse routed in the Tecnología Educativa community. Also, the research interests in digital competence and assessment and feedback differ from the other samples, and connect more closely to other backgrounds different from computing disciplines (mostly education sciences).

For both Germany and Spain, it is quite enlightening to see that education researchers seem to be anchored within their national and linguistic communities, whereas psychology and technology researchers, respectively, seem to integrate with the English-publishing community. This could in part be explained by the relative context-independence of experimental psychological research and technology and information science research, as opposed to locally bound education research (Berliner, 2002).

The UK context differs to that of Germany and Spain. As a research community—or communities—of TEL, the language of scientific communication is English, making translations of terms obsolete, with researchers utilising the already English-language shaped terminology. The most common UK keywords follow that found by Bond et al. (2019), with their content analysis of all journal articles published in the *British Journal of Educational Technology* between 2010 and 2018 revealing an expanded focus across research on the *learning* processes of students and the emergence of *learning analytics* as a way of understanding these processes. This also extends to ethical and privacy discussions around the use of analytics and, in particular, the institutional usage of student data. Also, it is noteworthy to note the major presence of research around two particular problematic issues of the use of technology in education: *internet addiction* and *cyberbullying*. This may deserve further study to understand these concrete issues, which were much more present in the UK context than in the other countries.

Whilst it was unsurprising that more UK authors published in the *British Journal of Educational Technology* than Spanish and German authors, given its home in the UK, authorship in the journal has become increasingly international in the past decade, particularly from Europe and North America, but also from Asia and Oceania (Bond et al., 2019), as has that of *Computers & Education* (Zawacki-Richter & Latchem, 2018). This is also somewhat reflected in the nationality of authors across the sample, with 69.5% of articles with a UK corresponding author, followed by China, Australia and the US. This reflects the study by Guo et al. (2016), who conducted a similar analysis of co-authorship within the field of EdTech for publications between 2000 and 2012 in the Web of Science. The fourth most frequently collaborating pair across all countries was the UK and the US. The co-authorship here also reflects the close geographical ties with Ireland, as well as Britain’s Commonwealth past and present.

### Limitations

The present study does not come without limitations that mainly stem from the document corpus used for the analyses, which is defined via English language and search parameters. English language abstracts from researchers affiliated with higher education institutions from three different countries constitute the source for analysis. Therefore, the research activities that occur in regional and linguistically delineated scientific communities (Spanish and German), are not considered. In order to more comprehensively depict the use of terms used in the respective communities, it would have helped to also document the potential differences between the Non-English and the English language communities. Whilst linguistic specifics are addressed in the discussion, overall, this constitutes a limitation and merits further attention in ensuing studies.

Furthermore, potential shortcomings lie in the terms used in the journal search in relation to the sources of the study sample, as they may not have considered other common keywords in the EdTech research field according to countries. Also, it needs to be acknowledged that only Web of Science indexed EdTech journals were considered, so adding journals in other indexes could have added further (and probably different) insights. Finally, with this study being an analysis of existing material in EdTech research, further primary research could also probe into some of the topics raised in this study, e.g., literal translations of terms, as well as further investigation of the reasons for the primary focus of education researchers to focus on their linguistic communities as opposed to international ones. Thus, with results from this study providing a first delineation of the topic, they can be further deepened and extended through more differentiated analyses.

## Conclusions

As previously argued, research in the field of EdTech does reflect different research interests in relation to the varying research traditions within Germany, Spain and the UK, despite being published in the English language. Also, different topical clusters were identified for the three countries, which also confirms previous findings (Marín & Zawacki-Richter, 2019). In spite of the fact that English is considered the lingua franca of research across most scientific disciplines, linguistically defined communities and publication structures play an important role in the dissemination of knowledge (Beigel, 2021). This needs to be acknowledged alongside the research topics as such, as well as separately considered in its systematic implications.

The Germanic, Latin European and Anglo cluster are distinct from one another; however, they are still similar in economic terms; being among those with high GDP-PPP and the highest rate of economic freedom (Ronen & Shenkar, 2013). Whilst the economic status is not under closer consideration in this study, it is nevertheless noteworthy as economic aspects may influence the development of research on EdTech, especially considering the need to acquire and maintain digital and online technologies, and could even diametrically differentiate from research in countries within cultural clusters with a low GDP-PPP and lower rate of economic freedom (e.g., see economical poverty considered as an obstacle in the evolution of EdTech in Latin American countries; Maggio, 2000). This should be considered in future research, not least in order to potentially work on questions of divergence and convergence in relation to research topics.

As implications of the study, the need is considered to establish ways of understanding the traditions behind research articles written in English by non-English language authors and build collaborative systems to illustrate nuances in this research. For instance, future work could investigate the use of disciplinary thesauri and relate to the different traditions, also including examples of research articles (e.g., Mihalko et al., 2003). Against the background of transferability of results of education research to different contexts (Berliner, 2002), publication language as well as local research and application practice need to be considered more prominently in order for research to yield true impact. Furthermore, now more than ever with the consideration of the impact of the Covid-19 pandemic on education, future perspectives should foster a common dialogue that enhance collaboration in the field that help to address challenging research topics that affect all the countries to find joint solutions, such as the digital divide, digital literacies for students and educators, or the commercialisation of EdTech and issues of data privacy and safety (Bond, 2020; Bond et al., 2021; Marín, 2022).

## Appendix 1

List of journals included in the study and percentage according to country sample:
